# Comparison of vascular remodeling between a bioresorbable poly-L-lactic acid scaffold and a bare metal stent: a 6-month angiography and intravascular ultrasound analysis in porcine iliac arteries

**DOI:** 10.1080/15476278.2026.2630543

**Published:** 2026-02-12

**Authors:** Keita Hayashi, Hideaki Obara, Naoki Fujimura, Yohei Masugi, Yasuhito Sekimoto, Kentaro Matsubara, Yuko Kitagawa

**Affiliations:** a Department of Vascular Surgery, Hiratsuka City Hospital, Kanagawa, Japan; b Department of Surgery, Keio University School of Medicine, Tokyo, Japan; c Department of Pathology, Tokai University School of Medicine, Kanagawa, Japan; d Department of Surgery, National Hospital Organization Tokyo Medical Center, Tokyo, Japan

**Keywords:** Bare metal stent, Bioresorbable scaffold, Igaki-Tamai stent, peripheral arterial disease, porcine model, vascular remodeling

## Abstract

Animal experimental studies involving the Igaki-Tamai stent (ITS), a bioresorbable poly-l-lactic acid scaffold, in peripheral arteries are limited, and existing studies evaluated only short-term (3-month) outcomes. This study compared arterial responses associated with the ITS and bare metal stent (BMS) over 6 months using intravascular ultrasound (IVUS) analysis and evaluated feasibility in porcine iliac arteries. Four miniature pigs underwent stent implantation with the ITS in the right iliac artery and the BMS in the left iliac artery. Follow-up evaluations at 6, 12, and 24 weeks included angiographic and IVUS analyses to assess neointimal hyperplasia, percent area stenosis (%AS), and percent in-stent volume obstruction (%VO). Histological analysis was performed to evaluate tissue injury and inflammation scores. At 6 weeks, the neointimal area did not differ significantly between the ITS and BMS groups (8.49 ± 2.10 mm² vs 13.47 ± 6.67 mm², *P* = .205). However, the ITS group exhibited a significantly smaller neointimal area at 12 weeks (6.87 ± 1.15 mm² vs 20.65 ± 10.99 mm², *P* = .050) and 24 weeks (5.20 ± 0.85 mm² vs 22.32 ± 12.03 mm², *P* = .042). %AS and %VO were significantly lower in the ITS group at all follow-ups. The ITS group showed reduced tissue damage (injury score: 0.80 ± 0.430 vs 1.74 ± 0.908, *P* < .001) and inflammation (inflammation score: 1.25 ± 0.516 vs 1.67 ± 0.832, *P* < .001) compared with the BMS group. The ITS was associated with reduced vessel injury, lower inflammatory response, and favorable luminal remodeling over 6 months in healthy porcine iliac arteries.

## Introduction

The clinical outcomes of endovascular treatments for occlusive peripheral arterial disease (PAD) have improved markedly with the advent of novel devices. Endovascular stenting mitigates the issues associated with early elastic recoil, residual stenosis, and flow-limiting dissection after balloon angioplasty^1^; however, in-stent restenosis remains an unresolved challenge. Moreover, the use of permanent metallic stents leads to late stent fractures and generates imaging artifacts. Additionally, the stent remaining in the vessel may be an obstacle to future treatment.^2^ The concept of leaving nothing behind appears to be the solution to these issues.

A bioresorbable scaffold (BRS) differs from a bare metal stent (BMS) in terms of its ability to degrade within the arterial wall after fulfilling its role as a stent.^3^ Consequently, the BRS is anticipated to preclude issues such as early elastic recoil and vascular patency maintenance and to facilitate active structural and functional remodeling of the treated segment over time.^4^ Current BRSs consist of either a polymeric scaffold or bioresorbable metallic stent, with poly-L-lactic acid (PLLA) being the most frequently used polymer in clinical devices. PLLA undergoes metabolic degradation to carbon dioxide and water via the Krebs cycle over approximately 12–18 months.^5^ The Igaki-Tamai stent (ITS; Kyoto Medical Planning Co., Ltd.) is a BRS made of PLLA that was first used for a human coronary artery in 2000.^6^^,^^7^ Since 2009, the peripheral ITS has been commercially available for PAD treatment in Europe.^8^ Nonetheless, while ITS placement in the coronary artery has many *in vivo* experimental results, only a few animal experimental studies involving the peripheral arteries have been conducted.^9^^,^^10^ Moreover, these studies have evaluated only short-term (3-month) outcomes, and no previously published data have presented detailed *in vivo* experimental results on ITS implantation in the peripheral arteries exceeding 3 months, including intravascular ultrasound (IVUS) analysis. Therefore, the biological mechanisms underlying vascular responses to the ITS in peripheral arteries remain incompletely understood.

The current study aimed to compare arterial responses between the ITS and BMS over 6 months using IVUS analysis in healthy porcine iliac arteries, in order to characterize mid-term histobiological responses and vascular remodeling under non-atherosclerotic conditions.

## Materials and methods

### Animals

Four 10-month-old male miniature pigs weighing approximately 35 kg were used. All animals received oral acetylsalicylic acid (200 mg/d) and ticlopidine (200 mg/d) as anti-thrombotic therapy from 2 d before stent implantation until the end of follow-up. The type and dose of anti-thrombotic agents were determined based on previous reports.^11^ All pigs were cared for in the animal research laboratory for at least 3 d prior to the procedure to acclimate them to the laboratory environment and were allowed free access to food and water before and after the procedure.

This study was approved by the Institutional Animal Care and Use Committee of the authors’ university under approval number A2022-233 and was conducted in accordance with the institutional guidelines and the ARRIVE guidelines. Animal care was provided by specialists, and the protocols adhered to the National Institutes of Health’s Guide for the Care and Use of Laboratory Animals (1996).

### BRS

The ITS is made of a non-drug-eluting, high-molecular-weight PLLA monofilament (molecular mass: 183 kDa) with a zigzag helical coil design similar to that of recent metallic stents. The scaffold is radiolucent and has two radiopaque gold markers at each end. A balloon-expandable system compatible with a 0.018-inch guidewire and a 7-Fr sheath was used for deployment. Balloon inflation was performed for 60 s at 10 atm (nominal pressure recommended by the manufacturer) when deploying the stent.

### Stent implantation

All procedures were performed under general anesthesia induced by intramuscular injection of 2 mg/kg midazolam and 0.1 mg/kg medetomidine and maintained with isoflurane using a respirator. The animals were placed in the supine position, orally intubated, and mechanically ventilated. After establishing an intravenous line, cefazolin sodium hydrate (1,000 mg) was administered intravenously as antibiotic prophylaxis. Arterial blood pressure, heart rate, electrocardiogram, and blood oxygen saturation were continuously monitored during the procedure. The carotid artery was surgically exposed, and a 7-Fr BRITE TIP introducer sheath (Cordis) was inserted in a retrograde manner. A heparin bolus of 5000 U was administered intra-arterially, followed by an additional bolus of 2000 U every hour to prevent blood coagulation.

First, digital subtraction angiography (DSA) was performed using a 4-Fr pigtail catheter, and the reference vessel diameter before stenting was measured with IVUS. Prior to angiographic and IVUS analyzes, nitroglycerin (0.2 mg) was administered intra-arterially to prevent arterial spasms. A 36-mm length of appropriately sized ITS was implanted in the right iliac artery with balloon inflation for 60 s at 10 atm (the nominal pressure recommended by the manufacturer). For comparison, a 40-mm length of appropriately sized self-expandable BMS (S.M.A.R.T.; Cordis) was implanted in the left iliac artery. After implantation, the BMS was dilated using the ITS-attached balloon for 60 s at 10 atm. DSA and IVUS were performed immediately after stent implantation. The implanted stent size was determined according to the findings of the preimplantation IVUS analysis, with a stent-to-artery ratio of approximately 1.1:1.

### Follow-up protocol

Specialists continuously monitored the animals’ health during follow-up. Follow-up procedures were performed at 6, 12, and 24 weeks after stent implantation. The animals were sedated, and anesthesia was induced as previously described. The carotid artery was surgically exposed, and a 5-Fr sheath was placed in a retrograde manner. DSA using a 4-Fr pigtail catheter and IVUS were performed after systemic heparinization. All animals were euthanized after the final follow-up at 24 weeks.

### IVUS analysis

IVUS analysis was performed using the Volcano s5 imaging system (Volcano Corp.) and a Visions PV catheter (Volcano Corp.). IVUS images were recorded from 5 mm distal to the distal stent edge to 5 mm proximal to the proximal stent edge with automatic pullback at 0.5 mm/s. All IVUS data were stored on DVDs for offline analysis and analyzed using an OsiriX DICOM viewer (Pixmeo).

The cross-sectional area (CSA) of each in-stent segment was measured at 0.5-mm intervals. The reference vessel luminal area was defined as the vessel lumen CSA 5 mm proximal to the proximal stent edge. The vessel lumen CSA before stenting, stent CSA immediately after stenting, and both vessel lumen CSA and stent CSA of the in-stent segments at each follow-up point were measured. Vessel lumen and stent volumes were calculated using Simpson’s rule for each CSA measurement. The neointimal CSA was calculated as stent CSA minus vessel luminal CSA. The neointimal volume was calculated as stent volume minus vessel luminal volume. Percent area stenosis (%AS) was calculated as the percent difference between the neointimal CSA and stent CSA. Percent in-stent volume obstruction (%VO) was defined as the percent difference in the neointimal volume and stent volume. The mean CSA of all cross-sections was calculated and defined as the luminal area. At the minimum luminal area (MLA), %AS and %VO were evaluated for each stent. Changes in the reference vessel luminal area, stent luminal area, and vessel luminal area over time were assessed. Each luminal area change was defined as the percent change at each follow-up compared with the area immediately after implantation.

### Histological analysis

Each stented iliac artery was harvested and pressure-perfused with 10% neutral buffered formalin at 100 mmHg immediately after euthanasia. Tissues were fixed in formalin for at least 48 h. Iliac artery segments were embedded in methyl methacrylate and sliced into 3 in-stent levels (proximal, middle, and distal). The sections were stained with hematoxylin and eosin and assessed under light microscopy. Histological analysis was performed by an experienced pathologist, who also determined the injury and inflammation scores.

The injury score was graded from 0 to 3 for each stent strut, as previously described.^12^ The mean injury score was calculated by summing the injury scores of all struts at the 3 in-stent levels for all 4 pigs, divided by the total number of struts. The injury scores were defined as follows: 0, intact internal elastic lamina (IEL) and compressed media without laceration; 1, lacerated IEL and typically compressed media without laceration; 2, lacerated IEL and media with intact but compressed external elastic lamina (EEL); and 3, lacerated EEL and typically large lacerations of media extending to the EEL, with coil wires sometimes residing in the adventitia.

Inflammation in each stent strut was scored from 0 to 3. The mean inflammation score was calculated using the same method as that employed for the mean injury score. The inflammation scores were defined as follows: 0, no inflammatory cells surrounding the strut; 1, minimal noncircumferential lymphohistiocytic infiltrate surrounding the strut; 2, localized moderate-to-dense cellular aggregate surrounding the strut non-circumferentially; and 3, circumferential dense lymphohistiocytic cell infiltration in the strut.^13^

### Statistical analysis

Continuous variables are expressed as mean ± standard deviation. All statistical analyzes were performed using SPSS software version 26.0 (IBM Corp.). Differences between the ITS and BMS were assessed using Student’s t-test. Statistical significance was set at *P* < .05.

## Results

Stent implantation and all follow-up procedures were successful, with no dissections, vessel injuries, or other complications occurring in all animals. Additionally, all animals remained healthy and survived up to the scheduled follow-up period. Only one BMS was excluded from analysis owing to stent occlusion at the 12-week follow-up; other BMSs and all ITSs were angiographically patent until the final follow-up.

### Angiographic analysis

**Figure 1.**
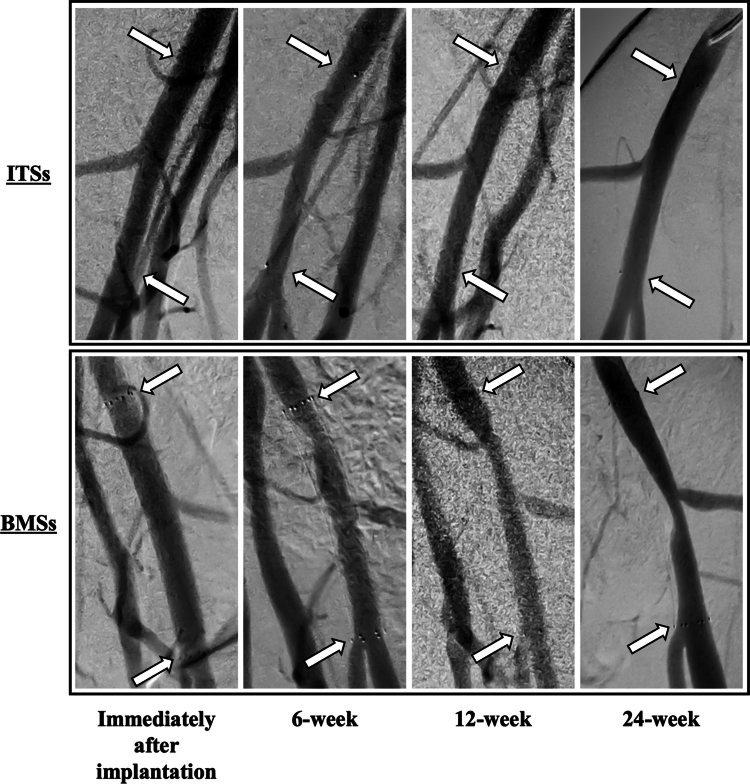
Representative digital subtraction angiography images of the same porcine iliac arteries for each scaffold immediately after implantation and at 6-week, 12-week, and 24-week follow-ups. Each arrow indicates an edge of the implanted scaffold. ITS, Igaki-Tamai stent; BMS, bare metal stent.

Representative changes observed on angiographic images over time in the same pig are shown in Figure 1. In the ITS group, maximum neointimal hyperplasia was observed at the 6-week follow-up; however, stenosis due to neointimal hyperplasia disappeared angiographically at the 24-week follow-up. In the BMS group, stenosis due to neointimal hyperplasia occurred at the 6-week follow-up and persisted at the 12- and 24-week follow-ups. Moreover, stenosis gradually worsened over time.

### IVUS analysis

**Figure 2.**
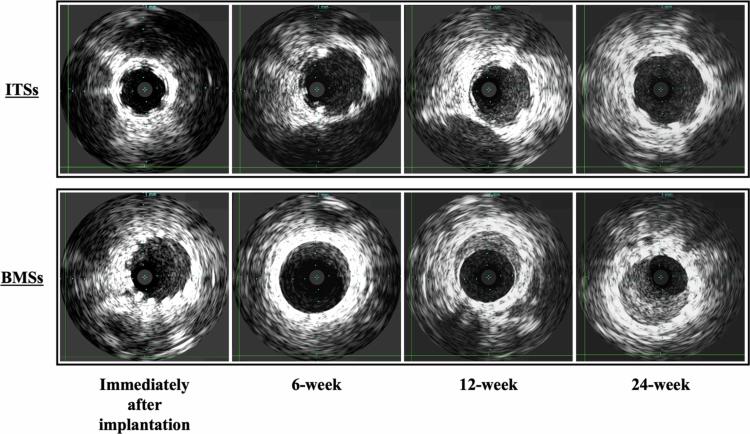
Representative intravascular ultrasonography images immediately after implantation and at 6-week, 12-week, and 24-week follow-ups. ITS, Igaki-Tamai stent; BMS, bare metal stent.

The pre-implanted vessel diameter, implanted stent diameter, and stent-to-artery ratio were similar in both groups (Table 1). Representative images of the IVUS analysis for each period are shown in Figure 2. Remarkable neointimal hyperplasia was observed at each follow-up period in the BMS group; conversely, this was not observed in the ITS group.

**Table 1.** Comparison of pre-implantation data for the ITS and BMS.

	ITS	BMS	*P* value
Pre-implanted vessel diameter (mm)	5.95 ± 0.44	5.65 ± 0.30	.305
Stent diameter (mm)	6.50 ± 0.58	6.25 ± 0.50	.537
Stent/artery ratio	1.09 ± 0.04	1.11 ± 0.03	.645

ITS, Igaki-Tamai stent; BMS, bare metal stent.

Baseline and follow-up IVUS data are presented in Table 2. The pre-implanted vessel luminal area did not differ statistically between the ITS and BMS groups (25.93 ± 4.65 mm^2^ vs 25.16 ± 3.26 mm^2^, *P* = .795). Moreover, no significant differences in the reference vessel luminal area and stent luminal area at each follow-up were noted between the 2 groups. The neointimal area also did not differ significantly between the ITS and BMS groups at the 6-week follow-up (8.49 ± 2.10 mm^2^ vs 13.47 ± 6.67 mm^2^, *P* = .205) but was significantly smaller in the ITS group at the 12- and 24-week follow-ups (12-week follow-up: 6.87 ± 1.15 mm^2^ vs 20.65 ± 10.99 mm^2^, *P* = .050; 24-week follow-up: 5.20 ± 0.85 mm^2^ vs 22.32 ± 12.03 mm^2^, *P* = .042). Furthermore, the neointimal area shrank over time in the ITS group; conversely, it expanded over time in the BMS group.

**Table 2.** Comparison of the results of intravascular ultrasound analysis between the ITS and BMS.

	ITS	BMS	*P* value
**Pre-implantation**			
Vessel luminal area (mm^2^)	25.93 ± 4.65	25.16 ± 3.26	.795
**6-week follow-up**			
Reference vessel luminal area (mm^2^)	30.39 ± 3.01	28.57 ± 2.68	.403
Stent luminal area (mm^2^)	23.06 ± 3.75	28.96 ± 3.73	.067
Neointimal area (mm^2^)	8.49 ± 2.10	13.47 ± 6.67	.205
**12-week follow-up**			
Reference vessel luminal area (mm^2^)	30.59 ± 1.49	28.54 ± 2.98	.279
Stent luminal area (mm^2^)	27.83 ± 5.26	30.68 ± 4.45	.485
Neointimal area (mm^2^)	6.87 ± 1.15	20.65 ± 10.99	.050
**24-week follow-up**			
Reference vessel luminal area (mm^2^)	37.84 ± 2.17	33.72 ± 1.10	.031
Stent luminal area (mm^2^)	32.85 ± 4.71	31.82 ± 5.33	.798
Neointimal area (mm^2^)	5.20 ± 0.85	22.32 ± 13.03	.042

ITS, Igaki-Tamai stent; BMS, bare metal stent.

**Figure 3.**
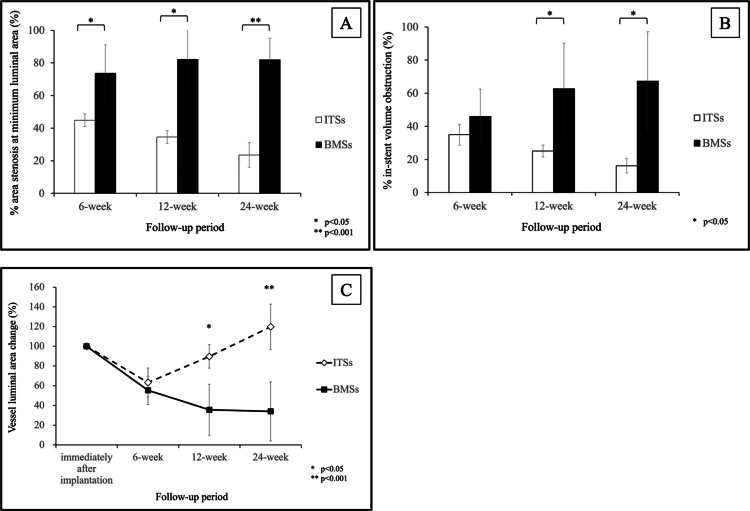
(A) Comparison of the percent area stenosis in the minimum luminal area between the Igaki-Tamai stent (ITS) and bare metal stent (BMS) at each follow-up. (B) Comparison of the percent in-stent volume obstruction between the ITS and BMS at each follow-up. (C) Changes in the vessel luminal area with the Igaki-Tamai stent (ITS) and bare metal stent (BMS). Data are presented as mean ± standard deviation. Comparisons between groups were performed using Student’s t-test. A *p* value < 0.05 was considered statistically significant.

Changes in %AS at the MLA are shown in Figure 3A. Throughout the follow-up period, %AS at the MLA was significantly lower in the ITS group than in the BMS group (6-week follow-up: 44.87 ± 3.96% vs 73.65 ± 17.42%, *P* = .042; 12-week follow-up: 34.56 ± 3.86% vs 82.23 ± 18.45%, *P* = .043; 24-week follow-up: 23.59 ± 7.54% vs 82.00 ± 12.23%, *P* = .001). %AS at the MLA showed symmetrical changes in the ITS and BMS groups.

Changes in %VO are presented in Figure 3B. At the 6-week follow-up, %VO was not significantly different between the ITS and BMS groups (34.96 ± 6.30% vs 45.96 ± 16.56%, *P* = .261). However, at the 12-week follow-up, the ITS group had significantly lower %VO than the BMS group (25.12 ± 3.59% vs 62.67 ± 27.6%, *P* = .039), and this difference remained significant at the 24-week follow-up (16.23 ± 4.35% vs 67.28 ± 29.91%, *P* = .018).

Changes in the vessel luminal area compared to each area immediately after stent implantation are shown in Figure 3C. The vessel luminal area shrank in both groups at the 6-week follow-up compared with baseline (63.22 ± 14.76% vs 55.32 ± 14.40%, *P* = .473). At the 12- and 24-week follow-ups, the vessel luminal area expanded over time in the ITS group (12-week follow-up: 89.65 ± 11.93%; 24-week follow-up: 119.68 ± 23.30%) but shrank over time in the BMS group (12-week follow-up: 35.64 ± 25.79%; 24-week follow-up: 34.03 ± 29.9%). A significant difference was observed between the ITS and BMS groups at 12 and 24 weeks.

### Histological analysis

**Figure 4.**
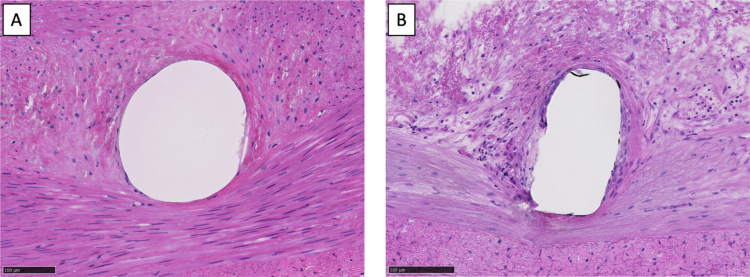
Representative photomicrographs of hematoxylin and eosin-stained sections of porcine iliac arteries at 24 weeks after stent implantation. (A) Igaki-Tamai stent (inflammation score: 1, injury score: 1). (B) Bare metal stent (inflammation score: 2, injury score: 2).

Representative photomicrographs of each stent strut are shown in Figure 4. The inflammation and injury scores were significantly higher in the BMS group than in the ITS group (inflammation scores [ITS vs BMS]: 1.25 ± 0.516 vs 1.67 ± 0.832, *P* < .001; injury scores [ITS vs BMS]: 0.80 ± 0.430 vs 1.74 ± 0.908, *P* < .001) (Table 3).

**Table 3.** Histopathological evaluation at the 24-week follow-up.

	ITS	BMS	*P* value
Inflammation score	1.25 ± 0.516	1.67 ± 0.832	<.001
Injury score	0.80 ± 0.430	1.74 ± 0.908	<.001

ITS, Igaki-Tamai stent; BMS, bare metal stent.

## Discussion

This study presents the mid-term observation of the histobiological and physiological responses to the ITS in the normal iliac arteries of miniature pigs over 6 months. The results of this study revealed that the ITS was associated with reduced neointimal hyperplasia, lower %AS and %VO, and improved luminal remodeling compared with the BMS. In addition, histological analysis demonstrated significantly lower vessel injury and inflammatory responses in the ITS group, suggesting favorable tissue compatibility in the mid-term.

Angiographic and IVUS analyzes showed that neointimal hyperplasia in the ITS group peaked at the early follow-up period and subsequently regressed over time, whereas progressive luminal stenosis persisted in the BMS group. The improvement in luminal area observed in the ITS group may be explained by several interrelated mechanisms. Neointimal hyperplasia is caused by the proliferation of vascular smooth muscle cells following vascular injury.^14^ Additionally, long-term stent expansion or overexpansion promotes neointimal proliferation owing to the stimulation of tissue injury.^15^ Unlike permanent metallic stents, the bioresorbable ITS does not exert sustained chronic radial force on the vessel wall. This absence of permanent scaffolding may reduce continuous mechanical stress, leading to less vessel wall injury and a diminished inflammatory response. Consistent with this concept, the present histological findings revealed significantly lower injury and inflammation scores in the ITS group. Reduced mechanical injury and inflammation may, in turn, suppress smooth muscle cell proliferation and extracellular matrix accumulation, thereby facilitating favorable vascular remodeling and late luminal enlargement. These mechanistic considerations may also help explain the differences between the present findings and previous reports on PLLA-based scaffolds.

Previous studies on the short-term outcomes of PLLA scaffolds reported that PLLA scaffolds showed greater neointimal thickening than BMSs; histomorphometric results also indicated that the higher inflammatory reaction of the vessel wall in PLLA affected neointima formation.^16-18^ Additionally, in the coronary arteries, late-acquired BRS malapposition and associated very-late scaffold thrombosis of the BRS have been recognized as issues.^19^ Regarding the ITS, Sekimoto et al. reported no significant difference in inflammation scores between the ITS and BMS groups on histopathological analysis at 6 weeks.^9^ In this study, the histological images of the ITS group showed that the stent strut remained within the intima, whereas in the BMS group, the IEL was excessively compressed and drained by the stent strut, with strong inflammation around the stent struts. Additionally, inflammation scores were significantly lower in the ITS group at the 24-week follow-up. This discrepancy may be attributable to differences in scaffold design and mechanical behavior. The ITS adopts a helical coil configuration and exhibits relatively low chronic radial force compared with conventional metallic stents, which may limit persistent vessel wall compression. In contrast to earlier-generation PLLA scaffolds with thicker struts or higher radial strength, these design characteristics of the ITS may contribute to improved long-term tissue compatibility. Although histological assessment of scaffold biodegradation was not specifically performed in this study, overt inflammatory reactions related to polymer degradation were not observed within the 6-month observation period.

The present findings should be interpreted in the context of the experimental model. Healthy porcine iliac arteries were intentionally used to isolate intrinsic vascular responses to the scaffold without the confounding effects of atherosclerosis, calcification, or heterogeneous plaque composition. This controlled design enabled a mechanistic comparison between the ITS and BMS with respect to vessel injury, inflammation, and remodeling. Nevertheless, these conditions differ substantially from human peripheral artery disease, where lesion complexity, calcification, and vessel recoil may strongly influence device performance. Concerning the clinical use of the ITS in peripheral arteries, both multicenter prospective observational and single-center retrospective studies have reported a poor ITS patency rate and a high target lesion revascularization rate, indicating that the ITS patency rate is not comparable to the existing BMS patency rate.^2^^,^^8^^,^^20^ Recently, Obara et al. reported the results of a multicenter prospective study of ITS implantation in the iliac lesion and a 5-year long-term data analysis.^21^ While ITS placement in human iliac arteries was confirmed to be safe, the primary patency rate at 12 months was 88.6%, which did not outperform the existing BMS patency rate. They suggested that these negative results in clinical use may be due to the low radial force of the ITS. These clinical observations underscore the importance of lesion characteristics and mechanical requirements when interpreting preclinical findings. The favorable vascular responses observed in this healthy animal model contrast with the inferior patency rates reported for the ITS in clinical studies involving atherosclerotic peripheral arteries.^2^^,^^8^^,^^9^^,^^20-22^ This apparent discrepancy likely reflects differences in lesion characteristics and mechanical requirements. In clinical settings, the relatively low radial strength of the ITS may predispose to early recoil when deployed in heavily diseased vessels. In contrast, the present study highlights the biological advantage of reduced chronic mechanical stress under non-atherosclerotic conditions, emphasizing that the current results provide mechanistic insights rather than direct predictions of clinical patency.

The difference in radial force between the ITS and BMS may also be viewed as an inherent design feature rather than a methodological bias. While the lower radial force of the ITS could contribute to reduced vessel injury and inflammation, this characteristic represents a fundamental property of bioresorbable scaffolds. From a mechanistic perspective, the present data suggest that minimizing chronic radial stress may be beneficial for vascular healing and remodeling in appropriately selected vessels. Furthermore, the advantages of bioresorbable devices are considered significant for cases requiring temporary scaffolding support as a bailout for dissections, as well as in pediatric cases where future vascular growth is anticipated.

Future investigations should focus on longer-term follow-up to assess vascular responses after complete scaffold resorption, evaluation in diseased or atherosclerotic models, and optimization of scaffold design, including potential drug-eluting modifications. There is currently no clinically available drug-eluting ITS; however, this project is in progress. Kuwabara et al.^10^ compared the results at 3 months between drug-free and drug-eluting ITSs in the iliac arteries of miniature pigs and reported a lower in-stent restenosis rate with the drug-eluting ITS. Such studies will be essential to clarify the appropriate clinical indications for bioresorbable scaffolds in peripheral arterial interventions.

This study has several important limitations. First, the follow-up period was limited to 6 months, which does not cover the complete biodegradation process of PLLA scaffolds. Consequently, vascular responses after full scaffold resorption and their effects on late vascular remodeling could not be evaluated. Second, scaffold biodegradation was not directly assessed, and biological responses associated with scaffold disappearance remain unknown. Third, the experiments were conducted in healthy porcine iliac arteries, and vascular responses in atherosclerotic lesions may differ from those observed in this model. Finally, the sample size was small, consisting of four animals. In addition, one BMS was excluded from analysis because of occlusion during follow-up, which limits the strength of statistical interpretation. The evaluation also relied mainly on morphometric and histological findings; incorporation of molecular or cellular markers related to endothelial healing and inflammation would allow a more detailed assessment of vascular responses. Therefore, studies with larger cohorts and longer follow-up periods are required.

In conclusion, implantation of the ITS in healthy porcine iliac arteries resulted in less vessel injury, reduced inflammatory response, and more favorable luminal remodeling over 6 months compared with a BMS. These findings indicate that PLLA-based bioresorbable scaffolds can achieve stable mid-term vascular remodeling in peripheral arteries under non-atherosclerotic conditions. Although direct extrapolation to clinical outcomes is not appropriate, the present results support further investigation of the ITS with respect to device optimization and appropriate clinical application.

## Data Availability

The authors confirm that the data supporting the findings of this study are available within the article.
